# Building Racial and Gender Equity into a National PrEP Access Program

**DOI:** 10.1017/jme.2022.37

**Published:** 2022

**Authors:** Jeremiah Johnson, Asa Radix, Raniyah Copeland, Guillermo Chacón

**Keywords:** Pre-exposure Prophylaxis (PrEP), HIV, Uninsured, Racial Equity, Gender Equity

## Abstract

Transgender and gender diverse (TGD), Black, and Latinx communities have long borne a disproportionate share of the U.S. HIV epidemic, yet these same key demographics are continually underrepresented in national PrEP prescriptions. Black, Latinx, and TGD individuals are also more likely to be uninsured, meaning that a proposed federal program to cover PrEP for people without insurance could provide significant benefit to potential PrEP users from these populations. However, coverage of PrEP costs alone will not end disparities in uptake. This commentary provides additional context and recommendations to maximize effectiveness of a national PrEP program for TGD, Black, and Latinx populations in the US.

The U.S. HIV epidemic is a case study of health disparities. While there have been reductions in incidence in recent years for white Americans, Black and Latinx communities have seen their epidemics stagnate or worsen. Black populations made up 29% of new infections in 1981 and 41% of new infections in 2019.^1^

Hispanic/Latino individuals made up 16% of new infections in 1981 and 29% of new infections in 2019.^2^ There are also high rates of HIV in transgender and gender diverse individuals. It is no coincidence that these same populations experiencing the highest number of HIV infections are disproportionately likely to be uninsured and are also much less likely to access PrEP therapy than white Americans.^3^ Recent CDC data found 17% of transgender women surveyed had no insurance.^4^ A plan must also factor in the challenges of addressing the needs of key populations across several different geographic settings, including following expert guidance to leverage telehealth for PrEP in rural areas which also experience significant racial and gender disparities in HIV outcomes.

There is no chance of success in addressing HIV in the United States without reducing disparities in coverage and PrEP access. It follows that any national PrEP access program, such as the one proposed by Killelea and colleagues,^5^ must consider and address unique barriers for the most affected communities from conception all the way through implementation with a constant focus on race and ethnicity, gender, sexual identify, and geographic settings including rural communities. Precedent exists for highly accessible PrEP programs in nontraditional settings that can disproportionately benefit communities that are generally excluded from interventions; however, previous successful examples highlight the importance of quality provider services and simultaneously addressing several financial, social, and structural barriers for priority populations.^6^ This commentary outlines specific considerations for Black communities, Hispanic/Latino communities, and transgender and gender diverse (TGD) communities.

## PrEP Access for Transgender and Gender Diverse People

The increased rates of HIV among TGD people, compared to the U.S. general population, are due to multiple contributing factors, but many have a foundation in the high rates of societal stigma and discrimination faced by TGD individuals.^7^ HIV screening and sexual health programs provide important entry points to PrEP; however, these are underutilized by TGD populations. Since 2006, the Centers for Disease Control and Prevention (CDC) has recommended that persons at high risk for HIV infection undergo HIV screening at least annually,^8^ but this has been found to be suboptimal among transgender men and women with only one-third ever screening for HIV.^9^ In addition to avoidance of health services due to experiences of or anticipation of discrimination,^10^ transgender individuals may not be adequately screened for extragenital STIs, which are often asymptomatic.

Additional factors related to low PrEP uptake include challenges related to health education and literacy including low rates of knowledge, low HIV risk perception, PrEP stigma and concerns about antiviral drug interactions with hormones and lack of inclusion in PrEP campaigns.^11^ There are limited studies that have evaluated PrEP persistence in TGD populations; however, one study revealed lower persistence among transgender women compared to cisgender men.^12^

Actions to increase PrEP uptake and persistence among TGD populations will require a multifaceted approach, including ensuring that medical systems and provider networks are welcoming to TGD individuals, clinicians are trained in culturally competent care, and that TGD persons at risk for HIV are provided with appropriately tailored information about HIV prevention interventions. HIV PrEP campaigns must be inclusive of and responsive to concerns of TGD persons, including explicit statements that PrEP will not decrease hormone levels.

Informed decision making about PrEP should include information about the available PrEP options, and a discussion about how to extrapolate results from studies that have been predominately conducted in cisgender populations to TGD people. One example is that event-driven PrEP has not been studied in TGD populations or in individuals assigned female at birth. Long-acting cabotegravir, studied in HPTN 083 and 084 did not include transgender men or individuals with known silicone or soft tissue fillers in the buttocks, which predominately impacts feasibility of cabotegravir injections for transgender women. In the future, it is important that all clinical trials of HIV prevention agents should be inclusive of TGD populations, including nonbinary persons, to assess effectiveness, feasibility, and acceptability.

## PrEP Access for Black Communities

The subpar utilization of PrEP among Black communities is symbolic of the ways America has lacked the will and capacity to effectively respond to the HIV crisis among Black communities. When PrEP was approved by the FDA in 2012, it was touted as a game-changing intervention for ending the HIV epidemic without a vaccine or cure. Yet, utilization among key populations in Black communities not only lags behind their racial peers, but dramatically widens the disparities of who will end their HIV epidemic by 2030 and those who will not. Black women are disproportionately impacted as well, making up 60% of new infections in women despite making up only 15% of women overall.^13^

Research has shown that barriers to PrEP utilization among Black communities include a multitude of racial and socio-economic factors including: lack of awareness; poverty; systemic racism; and culturally incompetent healthcare providers and systems.^14^ Black women in particular face challenges with awareness of PrEP, given that PrEP messaging is often not geared toward their particular messaging needs.^15^ Research points to effective structural interventions to respond to these intersectional issues that are driven by anti-Black systems. A national PrEP program that effectively reaches the most disenfranchised communities must respond not only to healthcare delivery issues, but long-standing racial and gender issues that span across various American systems that are traditionally viewed as non-healthcare issues. An effective program will be intersectional in its approach and address the factors that put some at higher risk of HIV acquisition than others.

A national PrEP program that effectively reaches the most disenfranchised communities must respond not only to healthcare delivery issues, but long-standing racial and gender issues that span across various American systems that are traditionally viewed as non-healthcare issues. An effective program will be intersectional in its approach and address the factors that put some at higher risk of HIV acquisition than others.

Access to quality healthcare is a facilitator of PrEP utilization. The first entryway to quality healthcare is universal healthcare coverage regardless of one’s ability to pay. While the proposal presented by Killelea and colleagues offers a pathway to immediate expanded and free access to comprehensive PrEP coverage, this is just one piece in the broader fight for comprehensive healthcare access. Providers in Black communities must also be trained to understand and ultimately believe in the efficacy and impact of PrEP in ending new HIV cases while also being continually engaged in a process that facilitates cultural humility to effectively reach Black individuals that would benefit from PrEP. Research in recent years has shown that acceptance of preventive healthcare services is more successful in Black patients when they are provided by Black providers and by programs designed by and for Black communities,^16^ something that likely factored into the successes of the PrEP demonstration project HPTN 073.^17^ Any national PrEP program would have to focus ongoing resources towards provider education and diversification of the healthcare and public health workforce in communities most impacted by HIV.

Health literacy and awareness about PrEP among Black communities continues to be an ongoing barrier nearly a decade after approval from the FDA. Health education and campaigns from CDC have not created broad awareness, and commercials from pharmaceutical companies have not led to a decrease in racial disparities among PrEP usage. Corporate campaigns may also have reinforced community beliefs that PrEP is something only gay men should use. The Black population of America is not monolithic; a national PrEP campaign must include wide-scale, culturally relevant marketing campaigns that respond to the concerns and motivations of a diverse group of populations within the Black community and also aim to reduce HIV stigma, homophobia, and transphobia. A national plan must also intentionally engage Black cisgender women who are not always aware that PrEP exists for them and often face high barriers when engaging clinicians for PrEP.

A national PrEP program must respond to structural issues that impact PrEP utilization along racial lines. Fundamental challenges like poverty, intimate partner violence, and a lack of housing all have deleterious effects on PrEP utilization. Structural interventions such as universal income and housing support have been shown to have positive impacts on health care seeking behavior. Linking PrEP access to programs that address these critical drivers of health disparities, and further strengthening participating organizations through financial incentives will better integrate PrEP into addressing the holistic wellness of Black communities most in need of comprehensive HIV prevention services.

## PrEP Access for Hispanic/Latinx Communities

As with TGD and Black communities, a broad range of socioeconomic and social barriers inhibit PrEP access in Latinx communities. Additionally, linguistic barriers and documentation requirements can make programs impossible to access for vulnerable Hispanic/Latinx individuals. A national PrEP access program that dramatically expands access points, nearly eliminates paperwork, and factors in culturally and linguistically relevant provider education and community promotion and health literacy will facilitate greater PrEP uptake in Hispanic/Latinx communities.

However, even as a national program is implemented, close monitoring and research will be urgently needed to better understand differential PrEP uptake among Black and Latinx communities with a particular focus on MSM. In studies that ask participants about PrEP interest and acceptability, reported willingness to adopt PrEP among Black and Latino MSM is as high (if not higher) than that reported by white and other MSM.^18^ However, hypothetical interest is not the same as real-world willingness, which is impacted not only by “objective” factors such as PrEP awareness/knowledge, but also by underlying attitudes and beliefs that influence comprehension and interpretation of any PrEP education.

In addition, PrEP *availability* is not the same as access. In order to be truly accessible, PrEP programs must be available to highest priority individuals within the settings in which they are most likely to receive care and in a manner that is financially affordable. As such, close monitoring and evaluation will help identify where and why PrEP access is lagging.

Emerging research also points to the important role of providers in PrEP implementation. Meaningful PrEP access for patients happens both before and after they express interest in PrEP.^19^ Many patients learn about PrEP for the first time from their medical providers, while others report that their providers have been a barrier to PrEP prescription. Comprehensive PrEP implementation requires increasing patient-provider communication about HIV prevention and ensuring that providers serving highest priority populations are willing and able to offer PrEP to them. Another recent study showed that community-based and culturally informed interventions can help overcome financial and insurance barriers to boost PrEP access among Latino or Hispanic sexual minority men.^20^

## Recommendations and Conclusions

To address racial, ethnic and gender inequities via a national PrEP access program, and building off of the innovative approach presented by Killelea and colleagues, at least a few common themes emerge:

1. *Intentionality:* a program must be designed specifically with racial, ethnic and gender equity in mind. Federal, state, and local implementers should continually consider if vulnerable Black, Latinx, transgender, and gender diverse communities are likely to benefit in rural, suburban, and urban contexts.
2. *Financial transparency and accountability:* program budgets should transparently account for the costs of a national PrEP program in a way that demonstrates that the intersectional needs of communities that need PrEP the most are being identified and properly resourced.
3. *Representation matters:* PrEP programs designed by and for the communities they aim to serve have been shown to be highly effective. In order to be strategic, any federal and health department leadership for a PrEP access program must reflect racially, ethnically and gender diverse communities. An expanded provider network must be shown to increase the number of Black, Latinx, and transgender providers offering services.
4. *Effective community outreach:* innovative messaging approaches must be pursued as knowledge of PrEP has not fully permeated vulnerable communities. Messages by and for current and potential PrEP users from priority populations will help reflect the key messages and address any linguistic barriers that prevent uptake of key information.
5. *Effective provider education:* In addition to receiving basic clinical guidance, any extended provider network established through a national PrEP access program must be effectively educated on unique barriers to uptake and access for Black, Latinx, and transgender populations.
6. *Socioeconomic factors:* PrEP access must be specifically paired with services that help to combat socioeconomic risk factors for vulnerable populations such as low income, unstable housing, and unsafe living environments. This should be considered in recruitment of a provider network, with specific emphasis on organizations that focus on these overlapping services across several geographic settings.
7. *Ongoing research:* Implementation of a federally coordinated access program should partner with NIH to study the effectiveness of scale up for Black, Hispanic/Latinx, and transgender populations, any persistent barriers to uptake, and potential extrapolation to other hard to access preventive healthcare services (e.g., Narcan). Implementation science must be prioritized to ensure we are documenting the successes and challenges of such a program.

## Note

Jeremiah Johnson provided consulting services to Johns Hopkins University during the conduct of the study.
